# Proteomic profiling of senescent human diploid fibroblasts treated with gamma-tocotrienol

**DOI:** 10.1186/s12906-018-2383-6

**Published:** 2018-11-29

**Authors:** Jen-Kit Tan, Faizul Jaafar, Suzana Makpol

**Affiliations:** Department of Biochemistry, Faculty of Medicine, Universiti Kebangsaan Malaysia (UKM), Jalan Yaacob Latif, Bandar Tun Razak, Cheras, 56000 Kuala Lumpur, Malaysia

**Keywords:** Gamma-tocotrienol, Replicative senescence, Human diploid fibroblasts, Proteomics

## Abstract

**Background:**

Replicative senescence of human diploid fibroblasts (HDFs) has been used as a model to study mechanisms of cellular aging. Gamma-tocotrienol (γT3) is one of the members of vitamin E family which has been shown to increase proliferation of senescent HDFs. However, the modulation of protein expressions by γT3 in senescent HDFs remains to be elucidated. Therefore, this study aimed to determine the differentially expressed proteins (DEPs) in young and senescent HDFs; and in vehicle- and γT3-treated senescent HDFs using label-free quantitative proteomics.

**Methods:**

Whole proteins were extracted and digested in-gel with trypsin. Peptides were detected by Orbitrap liquid chromatography mass spectrometry. Mass spectra were identified and quantitated by MaxQuant software. The data were further filtered and analyzed statistically using Perseus software to identify DEPs. Functional annotations of DEPs were performed using Panther Classification System.

**Results:**

A total of 1217 proteins were identified in young and senescent cells, while 1218 proteins in vehicle- and γT3-treated senescent cells. 11 DEPs were found in young and senescent cells which included downregulation of platelet-derived growth factor (PDGF) receptor beta and upregulation of tubulin beta-2A chain protein expressions in senescent cells. 51 DEPs were identified in vehicle- and γT3-treated senescent cells which included upregulation of 70 kDa heat shock protein, triosephosphate isomerase and malate dehydrogenase protein expressions in γT3-treated senescent cells.

**Conclusions:**

PDGF signaling and cytoskeletal structure may be dysregulated in senescent HDFs. The pro-proliferative effect of γT3 on senescent HDFs may be mediated through the stimulation of cellular response to stress and carbohydrate metabolism. The expressions and roles of these proteins in relation to cellular senescence are worth further investigations. Data are available via ProteomeXchange with identifier PXD009933.

**Electronic supplementary material:**

The online version of this article (10.1186/s12906-018-2383-6) contains supplementary material, which is available to authorized users.

## Background

Aging is a gradual decline in normal physiological process, often associated with age-related diseases which have negative impact on care and independence of elderly. The process of aging has been studied extensively to have a better understanding of its molecular basis in order to improve the quality of life among elderly. Human diploid fibroblasts (HDFs) cultured in vitro undergo a limited number of cell divisions due to the shortening of telomere which causes growth arrest, a phenomenon known as replicative senescence [1, 2]. Replicative senescence of HDFs has been used as a cellular model to study the mechanism of aging. High-throughput approaches at transcriptomic and proteomic levels have been used to identify the molecular changes in senescent fibroblasts. While both approaches complement each other, proteomics have the advantage that proteins are the final gene products which can be targeted for drug design. Proteomic profiling of differentially expressed proteins (DEPs) in young and senescent fibroblasts has been carried out using different methods such as two-dimensional gel electrophoresis (2DGE) [3, 4], iTRAQ labeling [5], two-dimensional differential gel electrophoresis (2D-DIGE) [6]. DEPs in young and replicative senescent cells include 90 kDa heat shock response protein (Hsp90), vimentin, collagen type I, α-enolase, annexin and RAN-specific-GTPase-activating protein [4, 5]. These findings indicate that many pathways are altered in senescent cells which may contribute to organismal aging.

The free radical theory of aging postulates that aging is due to accumulation of free radicals over time which damages macromolecules such as nucleic acids, proteins and lipids in the body [7]. Therefore, dietary supplements which possess anti-oxidative property may ameliorate cellular aging. Vitamin E is a group of lipid soluble antioxidants that is consisted of 8 members, namely α-, β-, γ- and δ-tocopherols; and α-, β-, γ- and δ-tocotrienols [8]. While α-tocopherol is well-studied, little is known about tocotrienols. Tocotrienol-rich fraction (TRF) has been shown to increase proliferation in senescent HDFs [9]. TRF reduces senescent-associated beta-galactosidase expression and modulates anti-oxidative enzyme activity in senescent HDFs [10]. TRF elongates telomere length and prevents cell cycle arrest in senescent HDFs [11]. One member of the tocotrienols family, gamma-tocotrienol (γT3), has been shown to increase proliferation of fibroblasts from young and elderly donors; and protects them from oxidative stress [12]. Our previous study shows that γT3 increases proliferation while prevents cell cycle arrest in senescent HDFs [13]. Transcriptomic analysis reveals that γT3 modulates expressions of genes involved in inflammation, apoptosis, protein transport and cell redox [14]. γT3 decreases protein expressions of p16^INK4a^, cyclin D1 and hypo-phosphorylated retinoblastoma (pRb) in senescent HDFs [15]. Although the pro-survival effect of γT3 on senescent HDFs has been reported, the global proteomic profile of senescent HDFs treated with γT3 remains unclear. Understanding the mechanisms of γT3 in promoting the proliferation of senescent HDFs may provide new insights for the modulation of γT3 during aging. Therefore, this study applied label-free quantitative (LFQ) proteomics to detect DEPs in young and senescent HDFs; and in vehicle- and γT3-treated senescent HDFs.

## Methods

### Primary culture of HDFs

Primary culture of HDFs was established from the foreskins of three 9- to 12-year-old boys. Written consents were obtained from their parents with Ethical approval code: FF-249-2012. The isolation, culture and passaging of HDFs were performed as described previously [15]. Briefly, epidermis of the skin was removed. Then, dermis was cut into small pieces, transferred into a tube with 0.03% collagenase type I solution (Worthington Biochemical Corp, Lakewood, NJ, USA) and incubated at 37 °C for overnight. Digested dermis was rinsed with phosphate-buffered saline and cultured in Dulbecco’s Modified Eagle Medium (Gibco, Waltham, MS, USA) with 10% fetal bovine serum (Gibco) at 37 °C in a humidified incubator with 5% CO_2_. When culture reached 80–90% confluency, cells were harvested by trypsin (Gibco) and serially passaged at 1:4 expansion. The young cells were defined as having population doubling (PD) < 10, while the senescent cells were at PD > 55. The proliferation rate, morphology and senescent-associated marker (β-galactosidase) of young, senescent and γT3-treated senescent HDFs have been characterized in previous study [13]. Senescent HDFs at PD > 55 express β-galactosidase with flattened cellular morphology, while γT3 increases cell proliferation and decreases cell cycle arrest of senescent HDFs. The comparisons were made between young and senescent cells; and between senescent cells treated with γT3 and vehicle.

5 × 10^5^ cells were seeded in a 100 mm petri dish and allowed to grow overnight. Then, the medium was changed with fresh medium for untreated young and senescent cells, with medium containing 70 μM γT3 or 0.1% ethanol (as vehicle) for senescent cells. After 24 h, the cells were harvested by trypsinization. Three biological replicates were included for each group. γT3 (> 96%) was obtained from Malaysian Palm Oil Board (MPOB, Selangor, Malaysia).

### Protein extraction

Cells were lysed in RIPA buffer (Sigma-Aldrich, St. Louis, MO, USA) mixed with protease inhibitor cocktail (cOmplete, mini, EDTA-free tablet form Roche, Basel, Switzerland). Protein concentration was measured by Bradford reagent (Bio-Rad Laboratories, Hercules, CA, USA) and stored in − 80 °C until ready to use.

### In-gel digestion

In-gel digestion of protein sample was performed according to method described previously [16]. Briefly, 50 μg of protein per sample was mixed with sample buffer (final concentration: 50 mM Tris-hydrochloride at pH 6.8, 2% sodium dodecyl sulfate (SDS), 10% glycerol, 0.1% bromophenol blue) and heated at 95 °C for 10 min. Protein sample was separated by SDS-polyacrylamide gel electrophoresis using a gel made up of 5% stacking gel and 12% resolving gel. After electrophoresis, the gel was washed with ultrapure water (Milli-Q water; Merck Millipore, Burlington, MA, USA) for 3 times at 5 min each. The gel was stained with Coomassie solution (SimpleBlue SafeStain; Invitrogen, Carlsbad, CA, USA) for 1 h at room temperature. After staining, the gel was washed with ultrapure water for 1 h and washed again with ultrapure water for overnight. Each gel lane (represented 1 sample) was cut into 6 fractions with approximately 1 cm length per fraction. Each fraction was cut further into smaller gel plugs of approximately 1 mm cube and transferred to a 2 mL tube. Reduction and alkylation of the gel plugs were performed as followed: incubated with 100% acetonitrile (ACN) for 10 min, 10 mM dithiothreitol in 100 mM ammonium bicarbonate (ABC) for 30 min at 56 °C then cooled to room temperature; followed by incubation with 55 mM iodoacetic acid in 100 mM ABC for 20 min in dark and finally incubated with 100% ACN for 10 min. The gel plugs were destained with 50% ACN in 100 mM ABC for 30 min followed by 100% ACN for 10 min. Digestion of the proteins in gel plugs was performed with 1.33 ng/μL trypsin (1:30 = trypsin:protein; Trypsin Gold, mass spectrometry grade from Promega, Madison, WI, USA) in 50 mM ABC and 10% ACN for overnight (> 16 h) at 37 °C. After the digestion, 5% formic acid:100% ACN (1:2, *v*/v) was added directly to the gel plugs and incubated for 10 min at 37 °C to extract peptides from the gel. Solution was transferred to a new tube and speed vacuumed for 3–4 h at room temperature. Dried peptides were stored at − 80 °C until use. All solutions were removed prior to addition of new solution and incubations were carried out at room temperature with shaking unless otherwise stated.

### Liquid chromatography tandem mass spectrometry (LCMS/MS)

All solvents were LCMS grade purchased from Fischer Scientific (Hampton, NH, USA). Peptides from each fraction were reconstituted with 15 μL mobile phase A (water/0.1% formic acid) and a 6 μL injection volume was drawn by Dionex Ultimate 3000 RSLCnano system (Thermo Scientific, Waltham, MA, USA) with C18 column (EASY-Spray LC column, 2 μm particle size, 75 μm D × 25 cm L; Thermo Scientific) heated at 55 °C. Gradient for nano/cap pump at a flow rate of 0.3 μL/min was set as followed: 5% mobile phase B (ACN/0.1% formic acid) at 0 min, 7% at 5 min, 25% at 90 min, 60% at 108 min, 95% at 113–123 min, 2% at 125–135 min. Peptides were ionized by electrospray ionization (EASY-Spray Source; Thermo Scientific) and analyzed by Q Exactive HF hybrid quadrupole-Orbitrap MS (Thermo Scientific). MS was operated at positive polarity with full MS scan (resolution: 120,000, 350–1800 *m/z*) followed by data dependent MS^2^ scan (resolution: 15,000, normalized collision energy: 28, isolated top 20 most intense ions).

### Protein identification and quantification

The mass spectral files (.RAW files) were processed using MaxQuant software (version 1.6.0.16) [17]. Default parameters were used for protein identification and quantification. Briefly, variable modification: methionine oxidation and N-terminal acetylation; fixed modification: cysteine carbamidomethylation; enzyme: trypsin; LFQ; false discovery rate: 0.01; and match between run were set for searching and quantification. FASTA file of human reference proteome was downloaded from UniProt (Proteome ID UP00000640; version January 2017) [18].

### Statistical analysis

The DEPs between groups were identified using Perseus software (version 1.5.4.1) [19]. LFQ intensities of proteins generated from MaxQuant were imported into Perseus. Data were pre-processed as following. Briefly, proteins identified as: only identified by site, reverse and contaminants were removed. Expression values were transformed to logarithmic scale with base 2. Samples were annotated according to their respective group. Valid values were filtered with minimum 70% presented in at least 1 group. The expressions of proteins between 2 groups were compared using 2-sample T-test with *p* value set at < 0.05. Principle component analysis (PCA) was performed on matrix before logarithmic transformation; multi scatter plot and histogram were constructed after filter of valid value; volcano plot was generated after 2-sample T-test; and hierarchical clustering was constructed for DEPs after calculation of z-score.

### Pathway analysis

DEPs were subjected to pathway analysis using Panther Classification System online (version 13.1) [20]. Briefly, IDs: protein IDs; organism: *Homo sapiens;* and analysis: functional classification viewed in gene list or pie chart, were set accordingly.

The mass spectrometry proteomic data have been deposited to the ProteomeXchange Consortium via the PRIDE [21] partner repository with the dataset identifier PXD009933.

## Results and discussion

### LFQ analysis

A total of 1878 proteins were identified through MaxQuant. After data pre-processing, 1217 proteins remained in young and senescent cells, while 1218 proteins for vehicle- and γT3-treated senescent cells. The entire dataset was provided in Additional file 1: Table S1.

PCA showed that the samples were clustered within group and separated with other group (Additional file 2: Figure S1). Multi scatter plot showed that each sample within the comparison was highly correlated with each other with Pearson correlation, *r* > 0.88; suggesting that no datasets were behaved as outliers (Additional file 2: Figure S2a & b). The datasets were normally distributed without obvious skewness as showed in histogram (Additional file 2: Figure S2c).

**Fig. 1:**
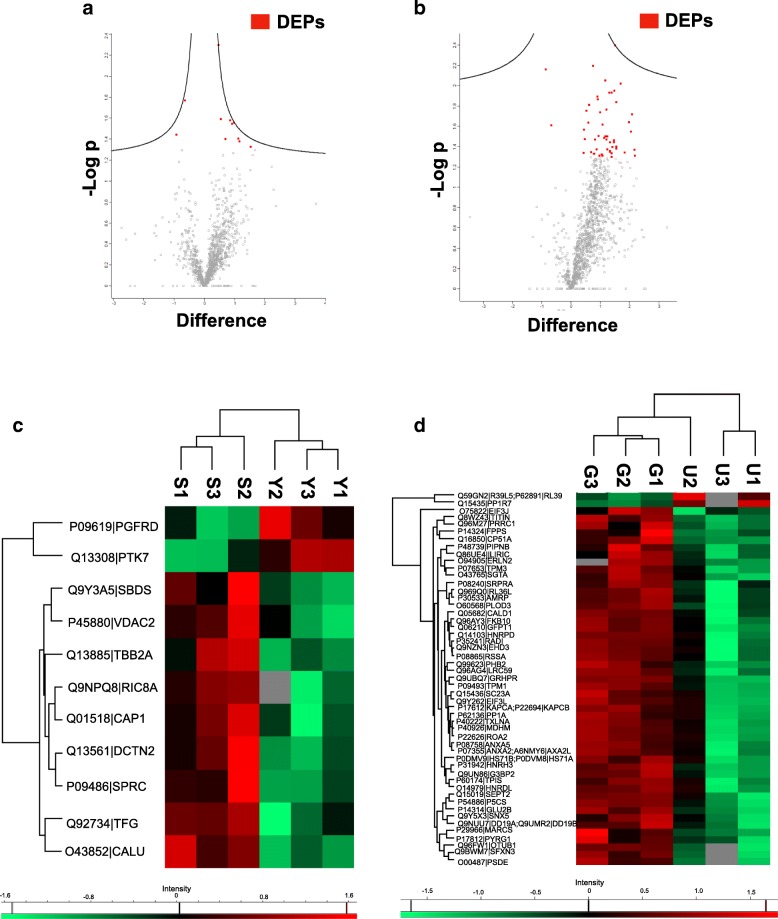
Graphical representations of differentially expressed proteins (DEPs). Volcano plot of total proteins for (**a**) young versus senescent cells and (**b**) vehicle- versus γT3-treated senescent cells. DEPs were highlighted in red. Heat map of DEPs for (**c**) young versus senescent cells and (**d**) vehicle- versus γT3-treated senescent cells. Y1–3: young cells; S1–3: senescent cells U1–3: vehicle-treated senescent cells; G1–3: γT3-treated senescent cells; *N* = 3 biological replicates for each group. Scale bar: young cells were set as control group for senescent cells; vehicle-treated senescent cells were set as control group for γT3-treated senescent cells

A total of 11 proteins were significantly expressed in between young and senescent cells (Table 1 and Fig. 1a & c), while 51 DEPs were found in between vehicle- and γT3-treated senescent cells (Table 2 and Fig. 1b & d).

**Table 1:** List of differentially expressed proteins in young and senescent cells

Protein ID	Protein name (symbol)
Upregulated	
Q01518	Adenylyl cyclase-associated protein 1 (CAP1)
O43852	Calumenin (CALU)
Q13561	Dynactin subunit 2 (DCTN2)
Q92734	Protein TFG (TFG)
Q9Y3A5	Ribosome maturation protein SBDS (SBDS)
P09486	SPARC (SPARC)
Q9NPQ8	Synembryn-A (RIC8A)
Q13885	Tubulin beta-2A chain (TUBB2A)
P45880	Voltage-dependent anion-selective channel protein 2 (VDAC2)
Downregulated	
Q13308	Inactive tyrosine-protein kinase 7 (PTK7)
P09619	Platelet-derived growth factor receptor beta (PDGFRB)

Young cells were used as a control group. Proteins were sorted according to alphabetical order

**Table 2:** List of differentially expressed proteins in vehicle- and γT3-treated senescent cells

Protein ID	Protein name (symbol)
Upregulated	
O00487	26S proteasome non-ATPase regulatory subunit 14 (PSMD14)
P08865	40S ribosomal protein SA (RPSA)
Q969Q0	60S ribosomal protein L36a-like (RPL36AL)
P30533	Alpha-2-macroglobulin receptor-associated protein (LRPAP1)
P40222	Alpha-taxilin (TXLNA)
P07355;A6NMY6	Annexin A2 (ANXA2); Putative annexin A2-like protein (ANXA2P2)
P08758	Annexin A5 (ANXA5)
Q9NUU7;Q9UMR2	ATP-dependent RNA helicase DDX19A (DDX19A); ATP-dependent RNA helicase DDX19B (DDX19B)
Q05682	Caldesmon (CALD1)
P17612;P22694	cAMP-dependent protein kinase catalytic subunit alpha (PRKACA); cAMP-dependent protein kinase catalytic subunit beta (PRKACB)
P17812	CTP synthase 1 (CTPS1)
P54886	Delta-1-pyrroline-5-carboxylate synthase (ALDH18A1)
Q9NZN3	EH domain-containing protein 3 (EHD3)
O94905	Erlin-2 (ERLIN2)
O75822	Eukaryotic translation initiation factor 3 subunit J (EIF3J)
Q9Y262	Eukaryotic translation initiation factor 3 subunit L (EIF3L)
P14324	Farnesyl pyrophosphate synthase (FDPS)
P14314	Glucosidase 2 subunit beta (PRKCSH)
Q06210	Glutamine--fructose-6-phosphate aminotransferase [isomerizing] 1 (GFPT1)
Q9UBQ7	Glyoxylate reductase/hydroxypyruvate reductase (GRHPR)
P0DMV9;P0DMV8	Heat shock 70 kDa protein 1B (HSPA71B); Heat shock 70 kDa protein 1A (HSPA71A)
Q14103	Heterogeneous nuclear ribonucleoprotein D0 (HNRNPD)
O14979	Heterogeneous nuclear ribonucleoprotein D-like (HNRNDL)
P31942	Heterogeneous nuclear ribonucleoprotein H3 (HNRNH3)
P22626	Heterogeneous nuclear ribonucleoproteins A2/B1 (HNRNPA2B1)
Q16850	Lanosterol 14-alpha demethylase (CYP51A1)
Q96AG4	Leucine-rich repeat-containing protein 59 (LRRC59)
P40926	Malate dehydrogenase, mitochondrial (MDH2)
P29966	Myristoylated alanine-rich C-kinase substrate (MARCKS)
Q96AY3	Peptidyl-prolyl cis-trans isomerase FKBP10 (FKBP10)
P48739	Phosphatidylinositol transfer protein beta isoform (PITPNB)
O60568	Procollagen-lysine,2-oxoglutarate 5-dioxygenase 3 (PLOD3)
Q99623	Prohibitin-2 (PHB2)
Q86UE4	Protein LYRIC (MTDH)
Q96M27	Protein PRRC1 (PRRC1)
Q15436	Protein transport protein Sec23A (SEC23A)
P35241	Radixin (RDX)
Q9UN86	Ras GTPase-activating protein-binding protein 2 (G3BP2)
Q15019	Septin-2 (SEPT2)
P62136	Serine/threonine-protein phosphatase PP1-alpha catalytic subunit (PPP1CA)
Q9BWM7	Sideroflexin-3 (SFXN3)
P08240	Signal recognition particle receptor subunit alpha (SRPRA)
O43765	Small glutamine-rich tetratricopeptide repeat-containing protein alpha (SGTA)
Q9Y5X3	Sorting nexin-5 (SNX5)
Q8WZ42	Titin (TTN)
P60174	Triosephosphate isomerase (TPI1)
P09493	Tropomyosin alpha-1 chain (TPM1)
P06753	Tropomyosin alpha-3 chain (TPM3)
Q96FW1	Ubiquitin thioesterase (OTUB1)
Downregulated	
Q15435	Protein phosphatase 1 regulatory subunit 7 (PPP1R7)
Q59GN2;P62891	Putative 60S ribosomal protein L39-like 5 (RPL39P5); 60S ribosomal protein L39 (RPL39)

Vehicle-treated senescent cells were used as a control group. Proteins were sorted according to alphabetical order

### Gene ontology analysis

**Fig. 2:**
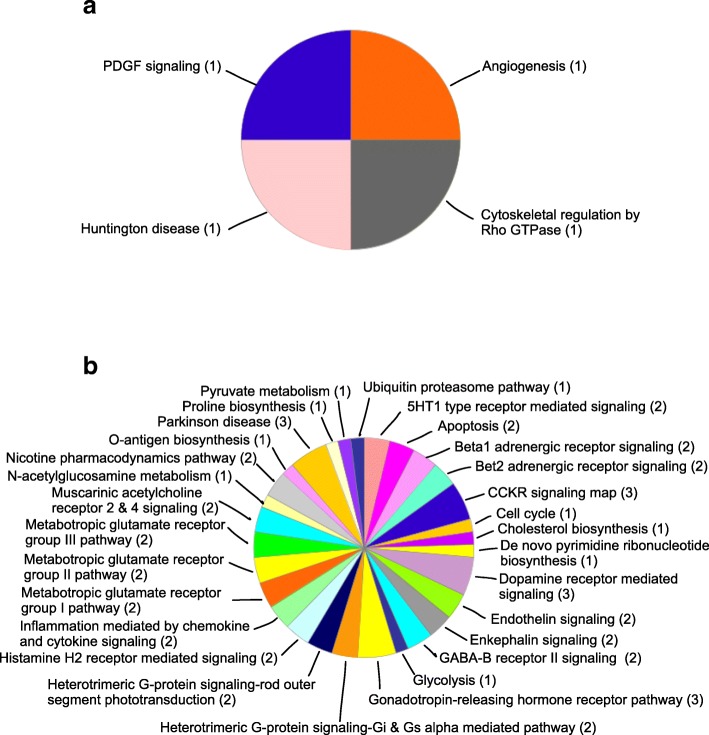
Distribution of pathways based on Panther Classification System. Pathways related to differentially expressed proteins in (**a**) young versus senescent cells and (**b**) vehicle- versus γT3-treated senescent cells. The number in bracket indicates number of proteins

Panther Classification System categorizes the DEPs based on their molecular function, biological process, cellular component, protein class (Additional file 2: Figures S3-S6) and pathway. The analysis showed that DEPs in between young and senescent cells belong to 4 categories of pathway which are angiogenesis, cytoskeletal regulation by Rho GTPase, Huntington disease and platelet-derived growth factor (PDGF) signaling pathway (Fig. 2a).

Based on the classification system, angiogenesis and PDGF signaling pathway are linked to PDGF receptor beta (*PDGFRB*) gene. PDGF is a mitogen for connective tissues such as fibroblast. It can be made up of homodimer of A, B, C and D polypeptide chains, and an AB heterodimer [22]. Its receptor, PGDFR is a type of tyrosine kinase receptor which activated upon ligand binding to form dimer, leading to a cascade of phosphorylation in pathways that promote cell growth, survival, migration and angiogenesis.

Two types of PDGFR, i.e. α and β receptors (PDGFRA and PDGFRB), exist which differ in ligand-binding specificity. PDGFRA binds to all PDGF chains except D chain, whereas PDGFRB binds only to B and D chains. Thus, different PDGF dimers can induce formation of αα, αβ or ββ dimeric receptor. *PDGFRB* mRNA level is lower in fibroblasts derived from patient with Werner syndrome (a premature aging condition) compared with normal subject, suggests that lack of PDGFRB expression decreases mitogenic response of cell to PDGF [23]. In contrast, PDGFRB protein is unchanged in senescent IMR-90 cell line compared with young cells, while decreased in cells after 4 weeks into senescence [24]. Similarly, there is no difference of PDGFRB protein expression in senescent and young cells of both human embryonic lung fibroblast IMR-90 and WI-38 cell lines [25]. Taken together, these findings suggest that PDGFRB protein expression may reduce only after a prolong culture of senescent cells. In this study, senescent cells were cultured for more than a week to be confluent before used for experiment. Our results showed that PDGFRB protein expression was downregulated in senescent cells compared with young cells. Our findings suggest that one of the mechanisms of replicative senescence may due to decrease level of growth factor receptor which leads to a reduced response to mitogenic stimuli and eventually diminishes the proliferative signaling pathways in senescent cells.

On the other hand, pathways in cytoskeletal regulation by Rho GTPase and Huntington disease are linked to tubulin beta-2A chain (*TUBB2A*) gene. β-tubulin interacts with mutant huntingtin protein which leads to impairment of intracellular transport [26]. Rho GTPase modulates dynamics and polarization of microtubules. Microtubules are mainly consisted of α- and β-tubulin heterodimers [27]. Protein level of tubulin beta-3 chain is higher in keratinocyte cells established from elderly donors than young donors, indicates that it may be a biomarker for skin aging [28]. In this study, TUBB2A protein expression was upregulated in senescent cells compared to young cells. However, the roles of TUBB2A in replicative senescence involving those two pathways are poorly understood.

For comparison between vehicle- and γT3-treated senescent cells in this study, the DEPs belong to 35 categories of pathway such as gonadotropin-releasing hormone receptor pathway, apoptosis signaling pathway, Parkinson disease, glycolysis and pyruvate metabolism (Fig. 2b).

Aging is associated with decrease in stress response which leads to accumulation of damaged proteins. Heat shock response is the primordial defense mechanism against stressful conditions. This response is modulated by a family of Hsp, such as 70 kDa Hsp (Hsp70), which assists in folding of new proteins and directs refolding or degradation of misfolded proteins that are often associated with neurodegenerative disease [29]. Hsp70 prolongs lifespan and improves cognitive function of aging mice [30]. In this study, Hsp70 was upregulated in γT3-treated senescent cells compared with vehicle-treated senescent cells. Our results suggest that γT3 may increase the stress response of senescent cells which accounts for its pro-survival effect on senescent cells as observed in previous study [15]. Cells isolated from aged organisms or undergoing replicative senescence have reduced heat shock response. Hsp70 protein level is higher in late passage of IMR-90 cells than early passage cells; and in fibroblasts isolated from elderly donors than young donors [31]. However, our results did not indicate a difference for Hsp70 expression in young and senescent cells. The reason for the discordant findings is unclear. In addition, due to the highly homologous sequences, Maxquant analysis could not differentiate members of Hsp70 family and could only annotate the detected protein group as either Hsp70-1a (gene name: *HSPA1A*) or Hsp70-1b (gene: *HSPA1B*) homolog. Further studies are needed to investigate the role of Hsp70 in senescence and determine which homolog is involved in the modulation using methods such as qPCR or immunoblotting with specific antibodies.

Caloric restriction (CR) has been shown as an effective way to prolong lifespan across many species, including human [32]. Carbohydrate metabolism is closely related to CR indicating the important role of energy metabolism during aging. Abnormal carbohydrate metabolisms are observed in cellular senescence which involve enzymes in glycolysis, tricarboxylic acid (TCA) cycle and malate-aspartate shuttle [33]. Triosephosphate isomerase (TPI) is a key enzyme in glycolysis and its deficiency causes accumulation of dihydroxyacetone phosphate which inhibits glycolysis [34]. Although it is a rare genetic disease, TPI deficiency is a progressive neurodegenerative condition closely resembles Alzheimer disease. TPI activity is decreased while its protein expression is unchanged with age in hippocampus of senescence accelerated mouse model, and increase of TPI activity by acupuncture intervention is accompanied with improvement of cognitive performance; suggesting that abnormal glucose metabolism is related to aging and dementia [35]. Our results showed that TPI protein expression was increased in senescent cells treated with γT3 compared with vehicle-treated cells, suggesting that γT3 may promote the glycolysis of senescent cells.

Malate-aspartate shuttle translocates NADH produced from glycolysis into matrix of mitochondria for ATP production via oxidative phosphorylation [36]. One of the key enzymes in this shuttle is malate dehydrogenase (MDH) which presents in two major forms: cytosolic MDH (MDH1) and mitochondrial MDH (MDH2), together they catalyze the interconversion of malate and oxaloacetate. MDH1 catalyzes the reduction of oxaloacetate to malate. MDH2, which is also part of TCA cycle, catalyzes the oxidation of malate to oxaloacetate. MDH1 activity and protein level are lower in senescent HDFs [37]. Furthermore, knockdown of MDH1 in young HDFs and IMR-90 cells induces senescence, while knockdown of MDH2 has no effect on cellular senescence of young HDFs. These findings indicate that MDH1 plays a critical role in regulating cellular senescence. In this study, MDH2 protein expression was higher in senescent cells treated with γT3 compared with untreated cells. Although MDH2 may not be directly regulating cellular senescence, it may still affect the energy metabolism as it is part of the shuttle. Increase of MDH2 protein level by γT3 suggests that γT3 may modulating the malate-aspartate shuttle and TCA cycle in senescent cells. Taken together, our results suggest that γT3 may promote survival of senescent cells by increasing carbohydrate metabolism that involves in glycolysis and malate-aspartate shuttle. However, one should be cautious when interpreting protein expressions of enzymes as their functionality would be better indicated by enzymatic activity. In addition, our DEPs results are different from previous studies [4, 5, 15] which show no change in expressions of proteins such as p16^INK4a^, cyclin D1 and pRb. This may due to the difference in (1) detection platforms such as immunoblotting, 2DGE, or label and unlabeled LCMS; (2) cell types used such as primary culture of HDFs or cell lines; and (3) post-translational modifications of protein such as performing phosphoproteome or whole proteome extraction. Further studies are required to investigate the role of these enzymes on energy metabolism during aging. Our previous study on transcriptomic profiling of senescent HDFs shows that γT3 upregulates mRNA expression of Hsp70 member 5 (gene: *HSPA5*) which involves in response to stress; and upregulates mRNA expression of mitochondrial alanine transaminase (gene: *GPT2*) which involves in carbohydrate and amino acid metabolisms [14]. Our transcriptomic and proteomic data show the same pathways are modulated by γT3 on senescent HDFs but the expression of mRNA is not in concordance with its protein expression. This may due to the difference in expression timing for mRNA and protein of the same gene. Future studies on integrating the data from transcriptomic and proteomic studies may provide more comprehensive view and new insights for understanding the mechanisms of cellular senescence and the modulation of senescence by γT3.

## Conclusions

LFQ proteomic analysis in this study showed that PDGFRB protein level was lower and TUBB2A protein level was higher in senescent HDFs than young cells, suggesting that pathways such as growth factor signaling and cytoskeletal regulation may be dysregulated during cellular senescence. On the other hand, protein levels of Hsp70, TPI and MDH2 were higher in γT3-treated senescent HDFs than vehicle-treated cells, suggesting that modulation of cellular senescence by γT3 may involve the stimulation of stress response and carbohydrate metabolism. The roles of these proteins in regulating cellular senescence worth further investigations.

## Additional files

Additional file 1: **Table S1.** Protein lists for the datasets. (XLSX 111 kb)

Additional file 2: **Figure S1-S6.** Showing the statistical and gene ontology analyses of the datasets. (PDF 443 kb)

## Abbreviations

2DGE: Two dimensional gel electrophoresis
ABC: Ammonium bicarbonate
ACN: Acetonitrile
CR: Caloric restriction
DEPs: Differentially expressed proteins
HDFs: Human diploid fibroblasts
Hsp: Heat shock response protein
LCMS/MS: Liquid chromatography tandem mass spectrometry
LFQ: Label-free quantitative
MDH: Malate dehydrogenase
PCA: Principle component analysis
PD: Population doubling
PDGF(R): Platelet-derived growth factor (receptor)
pRb: Phosphorylated retinoblastoma protein
SDS: Sodium dodecyl sulfate
TCA: Tricarboxylic acid
TPI: Triosephosphate isomerase
TRF: Tocotrienol-rich fraction
TUBB2A: Tubulin beta-2A chain
γT3: Gamma-tocotrienol
