# Supplementary material for: Proteomic profiling of senescent human diploid fibroblasts treated with gamma-tocotrienol
Source: BMC Complement Altern Med. 2018 Nov 29;18:314. doi: 10.1186/s12906-018-2383-6 (PMC6267793; doi:10.1186/s12906-018-2383-6)
Supplement: Supplementary file 2 — Figure S1-S6. Showing the statistical and gene ontology analyses of the datasets. (PDF 443 kb) [file 12906_2018_2383_MOESM2_ESM.pdf]

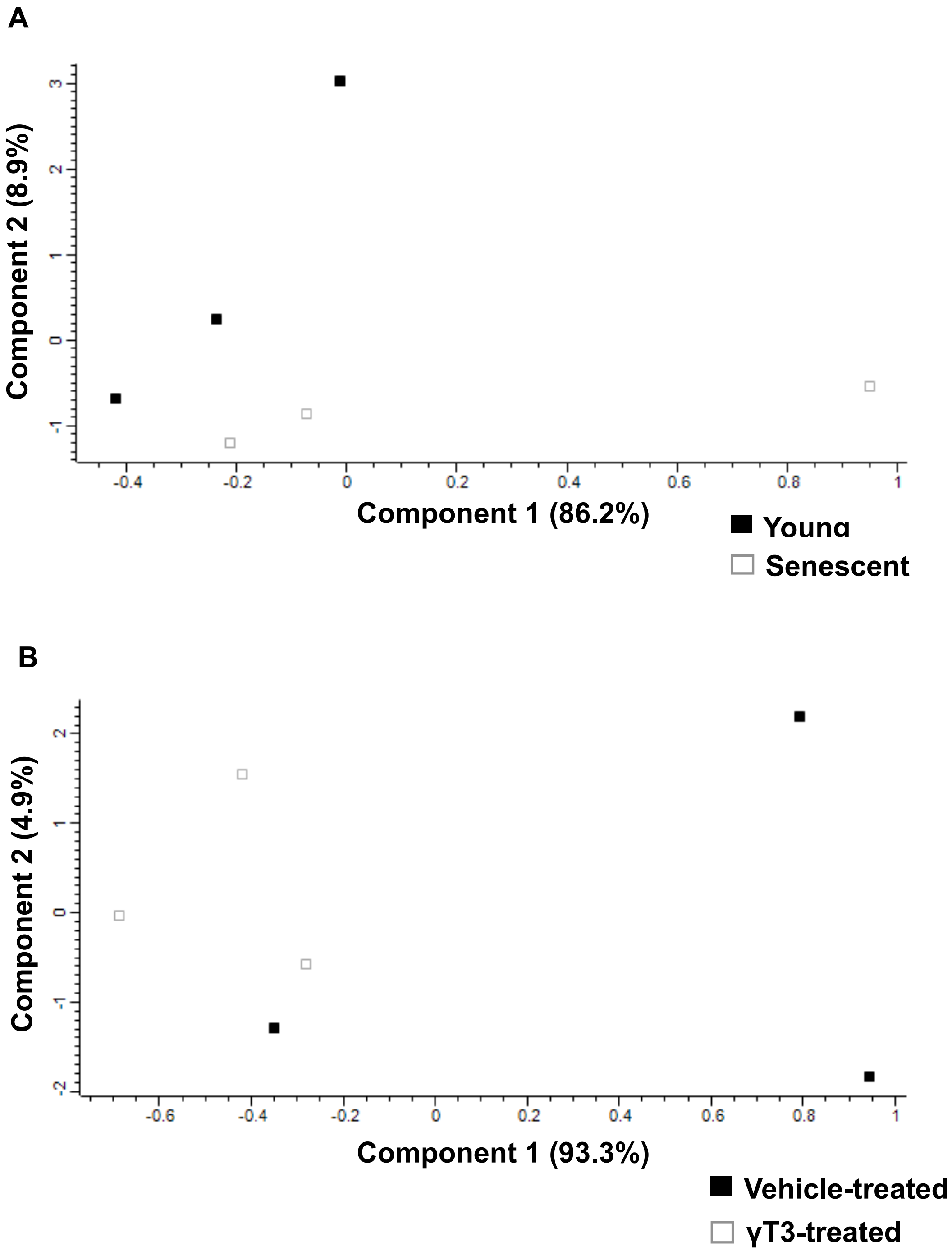

Figure S1 Principle component analysis of (A) young versus senescent cells and (B) vehicle- versus γT3-treated senescent cells. N=3 biological replicates for each group.

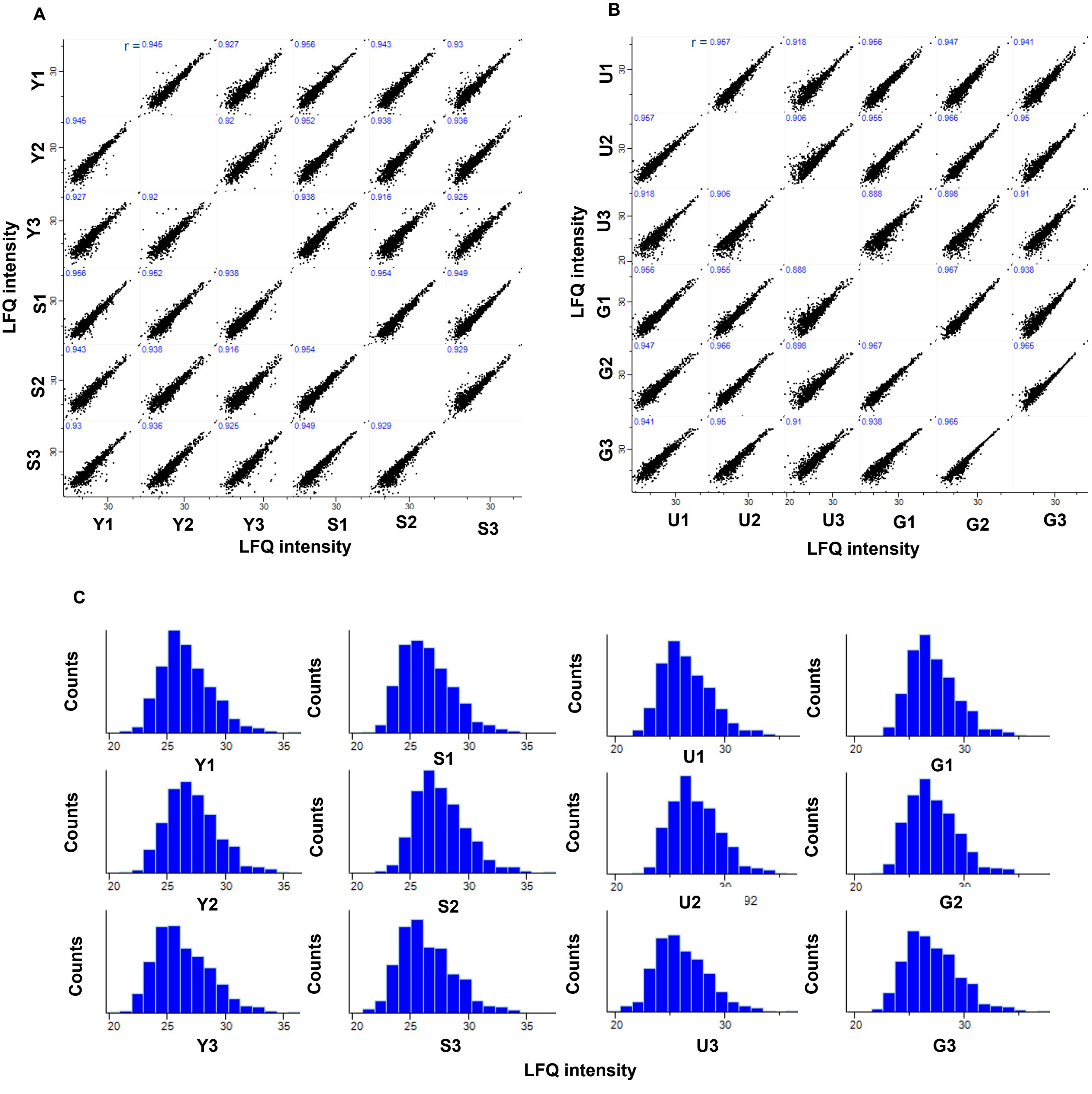

Figure S2 Quality control analysis of datasets. Multi scatter plot of datasets based on label-free quantitative (LFQ) intensity for (A) young versus senescent cells and (B) vehicle- versus γT3-treated senescent cells. Pearson correlation (r) was shown for each each analysis. Histogram of datasets based of LFQ intensity. Y1-3: young cells; S1-3: senescent cells; U1-3: vehicle-treated senescent cells; G1-3: γT3-treated senescent cells; each with 3 biological replicates.

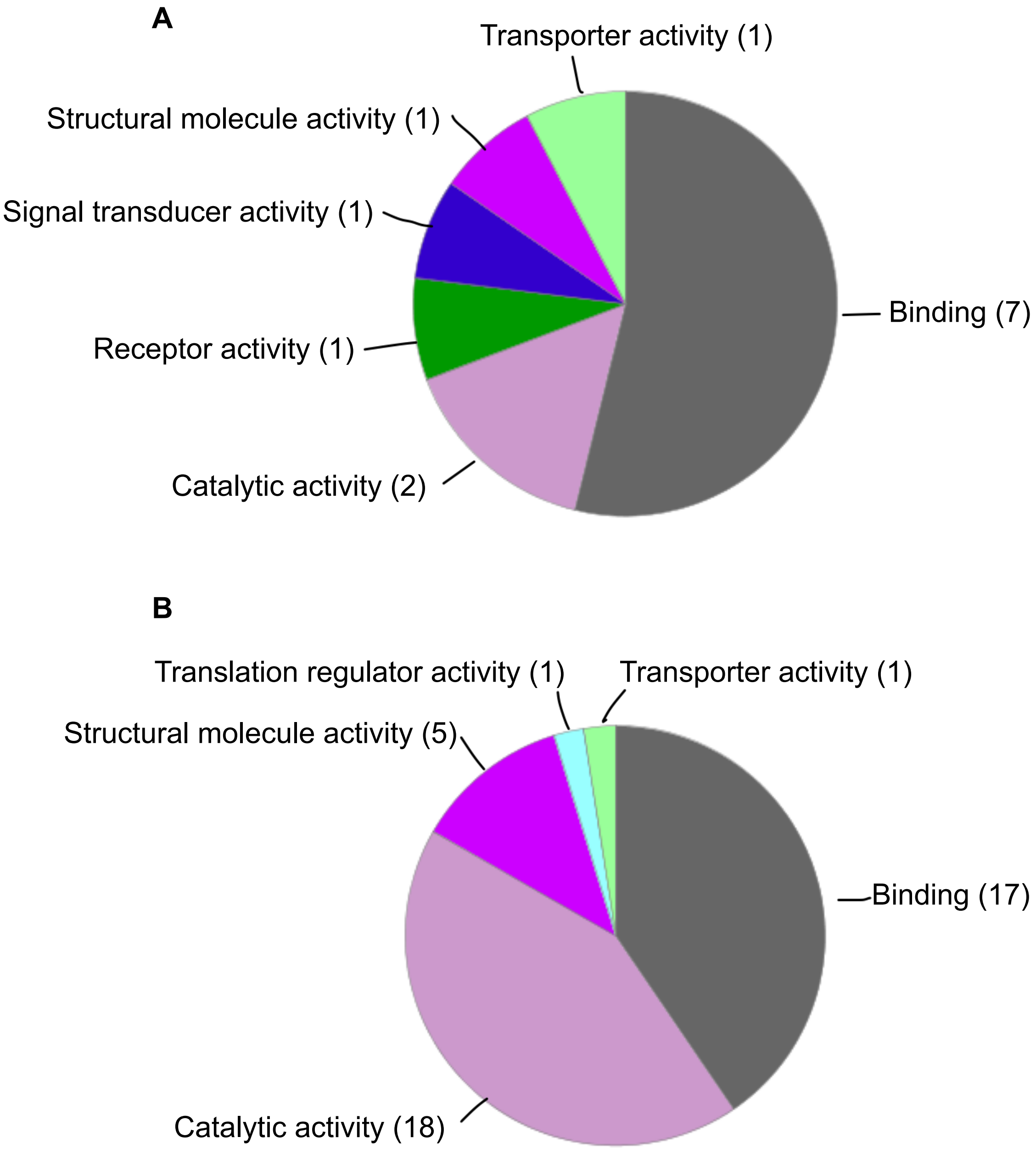

Figure S3 Molecular functions related to DEPs in (A) young versus senescent cells and (B) vehicle- versus γT3-treated senescent cells. The number in bracket indicates number of proteins.

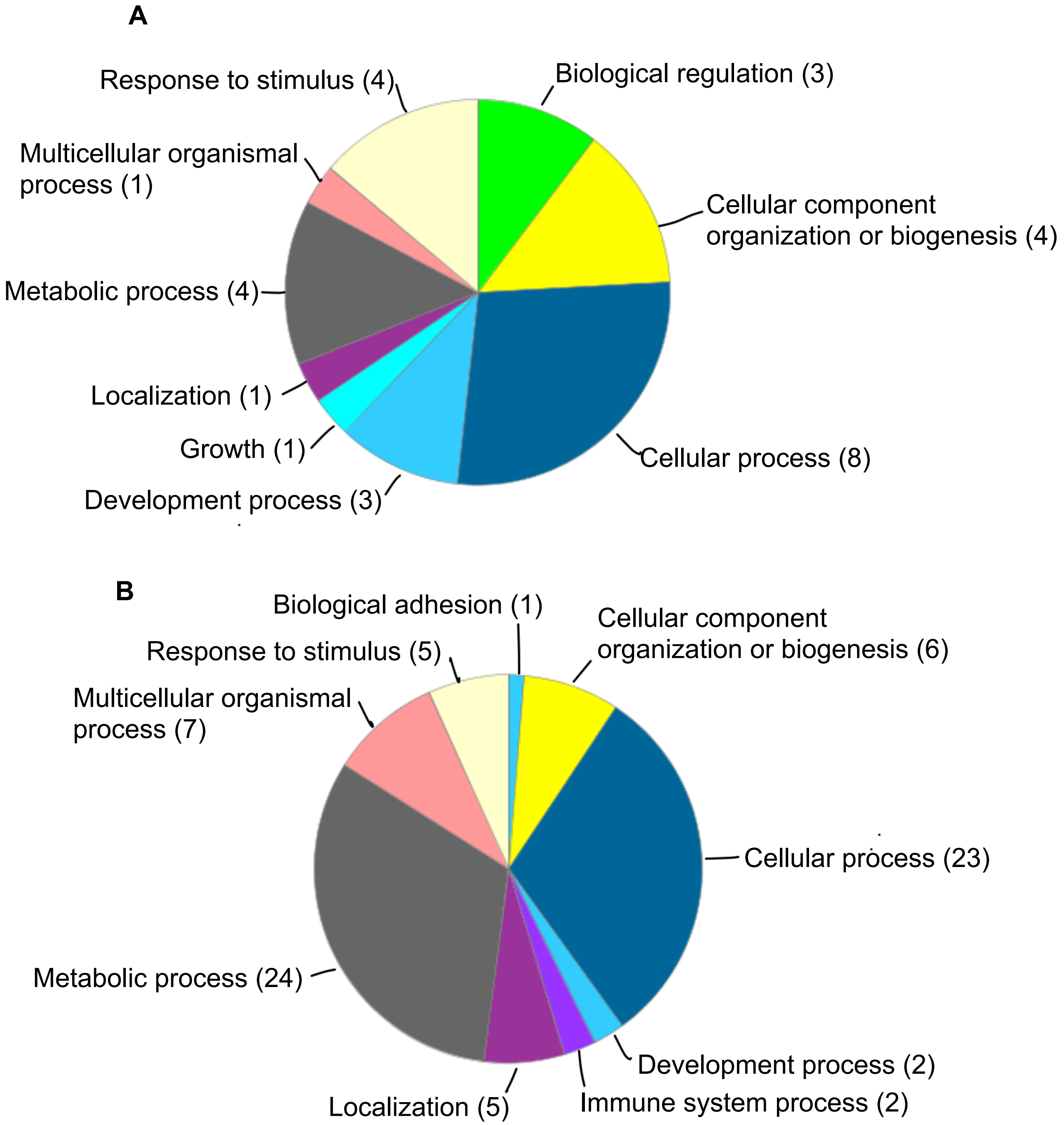

Figure S4 Biological processes related to DEPs in (A) young versus senescent cells and (B) vehicle- versus γT3-treated senescent cells. The number in bracket indicates number of proteins.

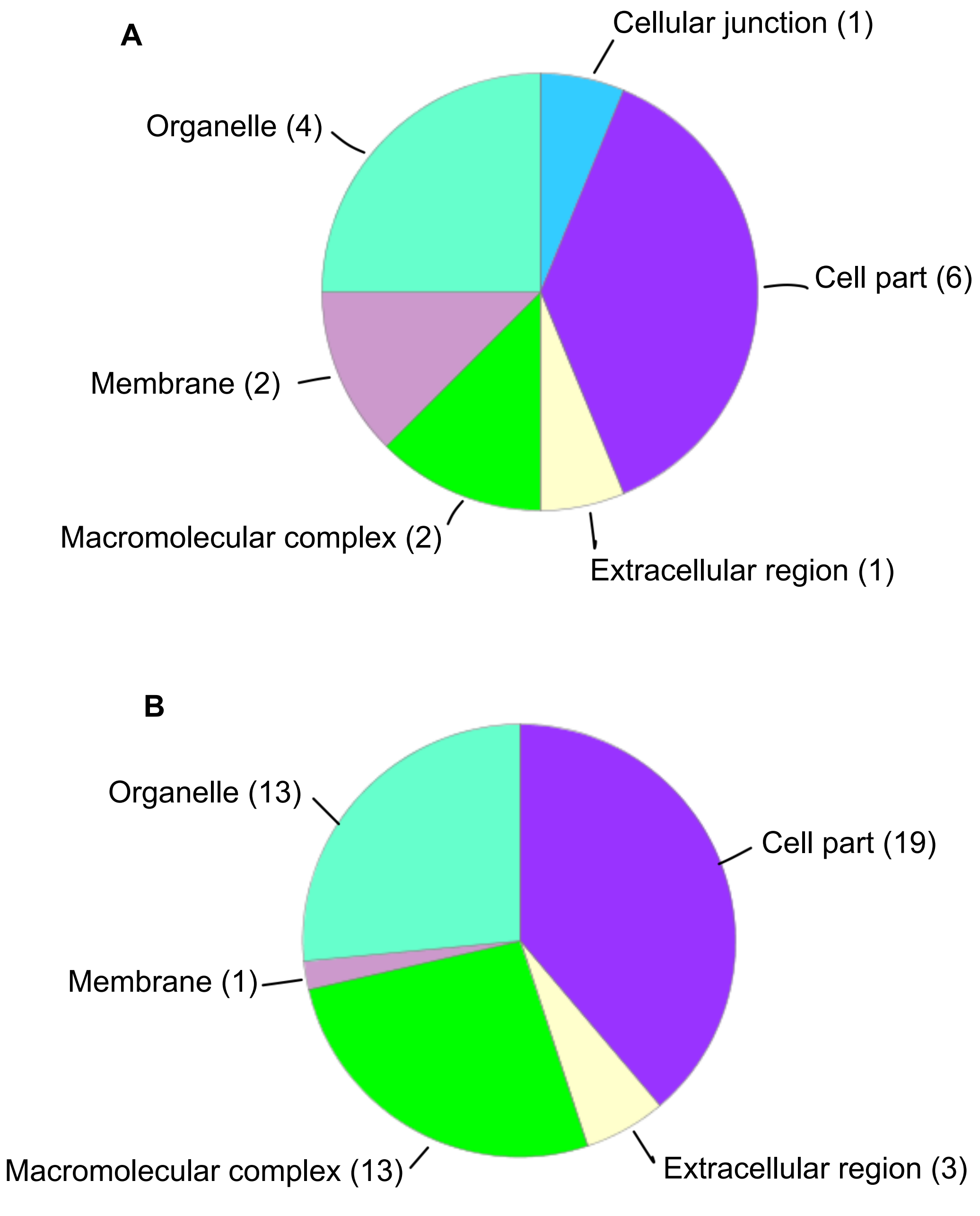

Figure S5 Cellular components related to DEPs in (A) young versus senescent cells and (B) vehicle- versus γT3-treated senescent cells. The number in bracket indicates number of proteins.

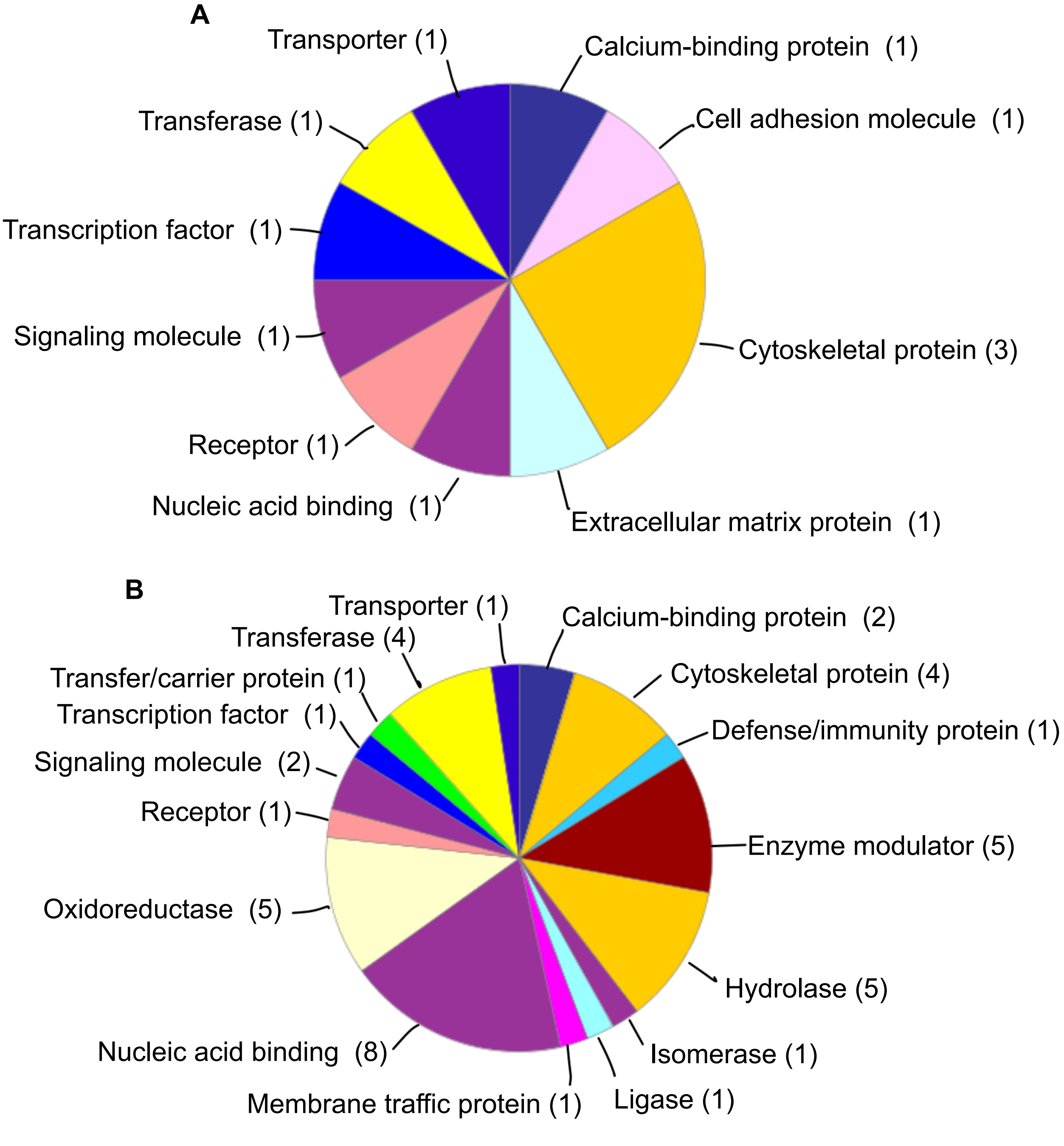

Figure S6 Protein classes related to DEPs in (A) young versus senescent cells and (B) vehicle- versus γT3-treated senescent cells. The number in bracket indicates number of proteins.
